# Old Dislocation of the Humerus

**Published:** 1848

**Authors:** H. S. Huber

**Affiliations:** Chicago


### ARTICLE III.

*Old Dislocation of the Humerus.* By H. S. Huber, M D., of Chicago.

Edward Quin, æt. 40, laborer, and a remarkably heavy built and muscular man, applied to me on the 1st of May, to relieve a pain in his left shoulder, which had been dislocated on the 29th of March, 33 days previously, by falling into the Canal, out of which he was wheeling a barrow of earth. He fell about five feet, striking his left shoulder against a large stone in the bottom of the excavation. A person who practised irregularly in the neighborhood attempted to reduce it, and told the patient it was all right.

I found the dislocation to be downwards and forwards, the head of the bone being under the edge of the great pectoral muscle. In consequence of the date of the injury, I omitted any endeavor to relax his system by the usual means, and attempted at once to replace it by means of the heel in the axilla.

The patient was extended on a bench, and the extending bandage applied above the elbow, to which I attached a folded sheet, crossed over my right shoulder; and with two men to assist the extension, it was kept up for about twenty minutes, without any perceptible good effect.

Dr. Bird was requested to give his assistance. A slit was cut in the centre of the folded sheet, just large enough to receive the injured shoulder, which was passed through it, so as to fix the scapula. This was attached to a hook secured into a door post; and to the extending bandage applied as before, we affixed a rope. Dr. Quinlan was desired to administer chloroform for the purpose of relieving the patient's suffering, whilst three men steadily exerted their strength in extending; the fore arm being flexed and used as a lever to rotate the head of the humerus, which we succeeded in drawing out a little. Upon adding a fourth man to the extending force, the loops which attached the arm to the rope, broke, after the extension had been applied about twenty minutes. The chloroform seemed to have the effect of partially relieving the pain, but otherwise it embarrassed the operation—exhilerating the patient, and causing him to endeavor to escape from the chair in which he was seated.

In order to keep up a regular and gradually increasing force, we now used the "twisted rope," which was a stout rope doubled, and attached to another hook in the opposite wall. Between its proximal end and the elbow, a swivel was interposed, the counter-extending apparatus was adjusted as before, the whole being drawn as tight as possible, and secured. A stick was inserted between the double rope, near the swivel, by which the rope was twisted, and gradully shortened; and after keeping up as great a degree of extension as the patient seemed able to bear for about a half an hour, with rotation of the humerus, &c., we sent for Dr. Brainard. Dr. B. thought there was as much strain as was prudent, and as so much extension had been employed, he thought he might reduce it by his heel in the axilla. Accordingly the apparatus was removed after having been used about an hour.

Dr. Brainard proceeded in the same manner described in my first attempt, his patient being on the floor, and having the arm at the same time freely rotated. During the extension the patient was bled to the amount of about xxx ℥, before he seemed to feel its effects; and although the head of the bone was somewhat more removed from its mal-position, it could not yet be replaced. This occupied about an hour.

It was determined to again employ the twisted rope. Dr. Maxwell was invited to witness the operation and agreed as to its propriety. The apparatus was arranged as in the first effort with this power, with the exception of the counter-extending bandage, which was passed altogether under the left arm, with a bandage attached to it, that crossed the top of the injured shoulder, and pressed against the acromion, which had the effect of better supporting the superior portion of the scapula.

Whilst the extension was in progress, ether was administered, but after a prolonged inhalation, it was found that he was but very slightly affected, and it did not seem to destroy his conciousness of pain. After about an hour, the arm being in the mean time freely rotated, the head of the humerous was found to have approached very nearly its normal position. A strong bandage was passed under the axilla, and over Dr. Brainard's shoulder, by which he endeavored to raise the head of the bone into the glenoid cavity; at the same time, Dr. Bird suddenly cut the connection between the extending rope and the elbow, whilst I forcibly drew the arm down against the anterior part of the patient's side, and the dislocation was found to be reduced.

This case illustrates the fact that great force may be applied if it is done steadily and gradually, the scapula being at the same time well fixed, without danger to the axillary artery. It is doubtless the tension that it is sometimes subjected to, by the scapula being drawn away from the thorax, that endangers its rupture.

I have referred so particularly to the "twisted rope," as it is a valuable substitute for the pulleys, which are not always at hand, especially in the country. The rope and its application are described and figured in the second edition of Fergusson's Surgery, except the swivel, which I found indispensable.

